# Emotional face recognition in male adolescents with autism spectrum disorder or disruptive behavior disorder: an eye-tracking study

**DOI:** 10.1007/s00787-018-1174-4

**Published:** 2018-06-19

**Authors:** C. C. A. H. Bours, M. J. Bakker-Huvenaars, J. Tramper, N. Bielczyk, F. Scheepers, K. S. Nijhof, A. N. Baanders, N. N. J. Lambregts-Rommelse, P. Medendorp, J. C. Glennon, J. K. Buitelaar

**Affiliations:** 1 Department of Cognitive Neuroscience, Donders Institute for Brain, Cognition and Behavior, Radboud University Medical Centre, Nijmegen, The Netherlands; 2 Centre for Cognition, Donders Institute for Brain, Cognition and Behavior, Radboud University, Nijmegen, The Netherlands; 3 Brain Center Rudolf Magnus, UMC Utrecht, Utrecht, The Netherlands; 4 Karakter Child and Adolescent Psychiatry University Centre Nijmegen, Nijmegen, The Netherlands; 5 Stichting Otto Gerhard Heldring, Zetten, The Netherlands; 6 Pluryn, Hoenderloo, The Netherlands; 7 Department of Developmental Psychology, Radboud University, Nijmegen, The Netherlands; 8 Department of Psychiatry, Donders Institute for Brain, Cognition and Behavior, Radboud University Medical Centre, Nijmegen, The Netherlands

**Keywords:** Autism spectrum disorder, Conduct disorder, Oppositional defiant disorder, Psychopathy, Eye-tracking, Callous and unemotional traits, Aggression

## Abstract

Autism Spectrum Disorder (ASD), Oppositional Defiant Disorder (ODD), and Conduct Disorder (CD) are often associated with emotion recognition difficulties. This is the first eye-tracking study to examine emotional face recognition (i.e., gazing behavior) in a direct comparison of male adolescents with Autism Spectrum Disorder or Oppositional Defiant Disorder/Conduct Disorder, and typically developing (TD) individuals. We also investigate the role of psychopathic traits, callous–unemotional (CU) traits, and subtypes of aggressive behavior in emotional face recognition. A total of 122 male adolescents (*N* = 50 ASD, *N* = 44 ODD/CD, and *N* = 28 TD) aged 12–19 years (*M *= 15.4 years, SD= 1.9) were included in the current study for the eye-tracking experiment. Participants were presented with neutral and emotional faces using a Tobii 1750 eye-tracking monitor to record gaze behavior. Our main dependent eye-tracking variables were: (1) fixation duration to the eyes of a face and (2) time to the first fixation to the eyes. Since distributions of eye-tracking variables were not completely Gaussian, non-parametric tests were chosen to investigate gaze behavior across the diagnostic groups with Autism Spectrum Disorder, Oppositional Defiant Disorder/Conduct Disorder, and Typically Developing individuals. Furthermore, we used Spearman correlations to investigate the links with psychopathy, callous, and unemotional traits and subtypes of aggression as assessed by questionnaires. The relative total fixation duration to the eyes was decreased in both the Autism Spectrum Disorder group and the Oppositional Defiant Disorder/Conduct Disorder group for several emotional expressions. In both the Autism Spectrum Disorder and the Oppositional Defiant Disorder/Conduct Disorder group, increased time to first fixation on the eyes of fearful faces only was nominally significant. The time to first fixation on the eyes was nominally correlated with psychopathic traits and proactive aggression. The current findings do not support strong claims for differential cross-disorder eye-gazing deficits and for a role of shared underlying psychopathic traits, callous–unemotional traits, and aggression subtypes. Our data provide valuable and novel insights into gaze timing distributions when looking at the eyes of a fearful face.

**Electronic supplementary material:**

The online version of this article (10.1007/s00787-018-1174-4) contains supplementary material, which is available to authorized users.

## Introduction

When communicating with others, non-verbal communication modalities such as body movements, hand gestures, and facial expressions yield essential information, in addition to verbal communication. Decoding facial expressions is one of the most efficient ways for understanding others’ emotions and feelings. Individuals with psychiatric disorders as Autism Spectrum Disorder (ASD), Oppositional Defiant Disorder (ODD), and Conduct Disorder (CD) exhibit deficits in regulating emotions and problems inhibiting aggressive tendencies [45, 46]. This may in turn explain dysfunctions in interpreting emotions of facial expressions. ASD are early onset neurodevelopmental disorders defined by core impairments in social interaction and verbal and non-verbal communication, stereotyped and restricted patterns of interest and activity, and abnormal sensory processing according to DSM-5 criteria [1]. ODD is characterized by angry and irritable mood, and argumentative, defiant, and disobedient behavioral patterns. CD is characterized by a pattern of aggressive, destructive, and/or deceitful behaviors that violate the rights of others according to the Diagnostic and Statistical Manual of Mental Disorders (DSM-5) criteria (APA, [1]. In this paper, we will combine ODD and CD into one diagnostic group, since both disorders are closely linked neurodevelopmental disorders of which ODD is either prodromal to CD or a subsyndromal form of CD [9]. The rationale for comparing these two distinct diagnostic cohorts (ASD versus ODD/CD) is that both involve social/communication problems and deficits in empathy (related to cognitive and emotional empathy, respectively).

In the latest version of the DSM-5, callous–unemotional (CU) traits were added as a specifier for a more severe form of CD labeled as having ‘limited prosocial emotions’ [1]. This form of CD is particularly associated with reductions in empathy when responding to fear, sadness, pain, and happiness of others [12]. ASD has also been associated with dysfunctional empathic functioning [1, 44, 73] and with increased levels of CU traits [55]. However, commonly deployed diagnostic questionnaires for ASD lack specificity to probe for CU traits, the relationship remains elusive. Nevertheless, empathy regulation is defined by two different constructs, namely (1) cognitive empathy (i.e., the ability to understand another’s feelings) and (2) emotional empathy (i.e., the experience of emotion, elicited by an emotional stimulus) [27].

Individuals with ASD often appear to have cognitive empathy deficits, but demonstrate average levels of emotional empathy [27, 49, 76]. In contrast, those with behavioral disorders (CD and ODD) show the opposite pattern (e.g., [16, 13]). Looking at facial emotion recognition from a behavioral perspective, no significant differences were detected when comparing ASD adolescent individuals to CD and TD individuals [51].

Eye tracking in ASD reports inconsistent findings regarding gazing at emotional faces. For an extensive meta-analysis and a summary of the reported differences during development, see [21, 41, 64]. The variation in reported results may partly be due to the variability in the methods utilised to study eye gazing in emotion recognition paradigms. Studies differ on their use of table-mounted remote eye-tracking devices or head-mounted ones. They also differ on their use of static and dynamic facial stimuli and the core characteristics of the faces (e.g., gender, intensity of emotions, and the appearance of the faces). In addition, many methodological issues cannot be properly controlled for which introduces additional heterogeneity. Studies differ in sampling frequencies of eye-tracking devices, the selection strategy of areas of interest, fixation classification filters, and the informed use of parametric or non-parametric statistical tests. Some of the earlier eye-tracking studies in adults and adolescents with ASD reported that less attention was paid to the eyes and other core features of faces [68] or focused more on the mouth and less on the eyes [53, 60]. Other studies confirmed that adults with ASD gazed less at the eye region while exploring a face [25, 43]. In contrast, more recent studies have not observed significant differences between individuals with ASD and typically developing youth in eye-gazing behavior [78, 82, 83]. More broadly speaking, gazing at the eyes can facilitate more accurate and faster responding to several emotions like fear, surprise, and disgust [6] and thus enable better social interaction. Numerous experimental studies have found strong evidence for reduced accuracy in identifying negative emotions in individuals with ASD [4, 7, 25, 48, 84], although there is no consensus in the field.

Overall, insufficient gazing to the eyes can lead to impaired emotional recognition which may influence disruptive behaviors and increase social anxiety in individuals with ASD [25].

Antisocial behavior is also associated with poor recognition and processing of fearful faces [56]. Recent studies confirm impaired recognition of multiple emotions (anger, fear, and happiness) in adolescents with CD relative to TD individuals [35, 36, 80]. Furthermore, children with greater behavioral problems (as indexed through the Psychopathy Screening device) also showed poorer recognition of angry, sad, and fearful facial expressions [10]. Those children and adolescents with both CD and high CU traits showed more pervasive impairments in emotional recognition than those with low CU traits [33, 36]. Recently, the first well-powered eye-tracking study on a large cohort of male and female adolescents with CD has been published. Martin-Key et al. [57] used an emotion recognition task with both static and dynamic morphed faces. They found that male adolescents fixated less on the eyes when viewing fearful and sad expressions. Although the differences were considered small, the authors suggest that behaviorally detected emotion recognition deficits were not mediated by abnormal fixation patterns [57].

ASD symptoms may moderate the relationship between CU traits and aspects of emotional empathy [70]. Pijper et al. [70] suggest that CU traits are inversely related to empathic sadness at low levels of ASD symptoms, while others document it only for higher levels of ASD symptoms [65]. Psychopathic traits seem to predict lower numbers of fixations and fixation durations to the eye region in fearful faces in TD male adolescents [29]. Individuals with ASD also have elevated levels of aggressive behavior compared to TD individuals [47], although aggression is not a core symptom of ASD and is typically less severe in ASD than ODD/CD [3]. For CD and ODD, both proactive and reactive aggression are considered hallmarks of the disorder [17], and the relation of subtype of aggression and eye-tracking patterns of emotional face processing is unclear. From a broader perspective, it seems that many concepts (i.e., psychopathic traits, CU traits, and subtypes of aggression) in different disorders (i.e., ASD, ODD, and CD) seem to be interlinked and associated with each other, while actual direct links remain elusive and a direct comparison is missing.

In summary, eye-tracking data in the literature related to emotional face processing are inconsistent in ASD and studies have not been properly replicated in large well-phenotyped psychiatric cohorts for CD and ODD. These relationships still remain elusive and the field suffers from inconsistency in approach to data collection and analyses and using fairly small sample sizes [41]. Our relatively large cohort (total *N* = 122; ASD = 52, ODD/CD = 42, TD = 28) consisting of male adolescents enables us to examine the common and unique eye-tracking patterns of emotional face processing in individuals with either ASD, ODD, or CD, in comparison with TD, and explore the possible modulatory role of CU traits, psychopathic traits and subtypes of aggression. We hypothesize that high CU traits, high psychopathic traits, and heightened proactive and reactive aggression will be associated with less time spent to the eye region for negative emotions (e.g., sadness, fear, and anger) in both male adolescents with ASD or CD/ODD. Furthermore, we hypothesize that both male adolescents with ASD and ODD/CD will show similar differences on the time to first fixation to the eye region of an emotional face.

## Methods

### Recruitment of participants

Initially, 423 individuals were approached to participate in a larger study on empathy (CU2 study). Individuals with an ODD/CD diagnosis were approached via institutes specialized in severe juvenile psychiatric problems (Karakter, Child and Adolescent Psychiatry) or severe disruptive behavior problems (De Hoenderloo Group, Otto Gerhard Heldring Foundation, and Woodbrookers). Individuals with ASD were recruited via information leaflets that were sent to families by the Dutch federation of Autism (NVA). The typically developing individuals’ control groups were recruited via leaflets that were sent to a community sample. These individuals were selected on the basis of their geographical location. The recruitment period lasted from April 2011 to September 2014. Of those approached, 265 did not respond or were not interested to participate. Of the 158 that were interested in participation, 18 did not meet the inclusion criteria (see below for more information). Two participants did not obtain consent from a legally appointed guardian and 6 participants were not able to participate due to their personal situation. In total, 132 were included for the broader CU2 study. Of the 132 participants, 6 participants did not undergo the extensive eye-tracking battery. Of the 126 participants, 4 participants had to be excluded based on exclusion criteria for eye-tracking data quality. Thus, all the presented data are from the 122 participants (50 with ASD, 44 with ODD or CD and 28 TD individuals). All participants were male adolescents [age range (12–19 years old, mean age= 15.26 years, SD = 1.9].

Main participant and demographic characteristics are summarized in Table 1.

**Table 1:** Characteristics of the study population (*N* = 122)

	Total group		TD		ODD/CD		ASD		Contrasts
	*M*	± SD	*M*	± SD	*M*	± SD	*M*	± SD	
Age (years)	15.2	1.9	15.9	1.8	15.2	1.7	14.9	2.0	n.s.
FSIQ	101.1	10.5	106.3	9.5	94.9	6.9	103.4	11.1	ODD/CD < ASD = TD***
VIQ	101.9	13.6	108.5	12.9	92.1	11.1	104.8	12.0	ODD/CD < ASD = TD***
PIQ	101.1	14.0	105.4	15.1	96.8	11.4	101.6	14.4	ns
ICU total scores									
ICU self-rated	26.8	8.8	23.6	6.3	31.0	10.0	24.9	7.6	ODD/CD > ASD = TD***
ICU parent-rated	28.8	11.3	17.0	7.1	38.9	7.7	28.1	8.3	ODD/CD > ASD > TD***
YPI self-rated scores									
Total score	93.55	23.1	82.21	17.1	106.87	23.2	88.45	20.6	ODD/CD > ASD = TD***
CU subscale	27.22	6.7	23.75	5.3	30.80	6.9	26.09	5.8	ODD/CD > ASD = TD***
RPQ self-rated scores									
Total score	13.46	8.4	7.64	4.3	19.30	8.4	11.31	7.0	ODD/CD > TD = ASD***
Reactive	9.54	5.2	6.00	3.3	12.30	4.8	8.84	5.1	ODD/CD > ASD*** > TD*
Proactive	3.79	3.9	1.64	1.8	6.71	4.6	2.34	2.5	ODD/CD > TD = ASD***
SCQ	11.38	7.1	3.96	3.1	11.24	6.0	15.64	5.8	ASD > ODD/CD < TD***

	Total group		TD		ODD/CD		ASD		Contrasts
	*n*	%	*n*	%	*n*	%	*n*	%	
	122	100.0	28	23.0	44	36.0	50	41.0	
Institute									
Child and adolescent psychiatry	50	41	0	0	9	20.5	41	82	
Youth welfare	36	30	0	0	35	79.5	1	2	
Dutch association for autism	8	6	0	0	0	0	8	16	
Comorbidity									
ADHD	50	41	0	0	28	63.6	22	44	ODD/CD > ASD > TD**
None	64	52	26	93	10	22.7	27	54	
Missing	9	7	2	7	6	13.7	1	2	
Medication									
Yes	48	55.8	0	0	17	38.6	31	62.0	ASD > ODD/CD > TD*
No	68	39.3	28	100	21	47.8	19	38.0	
Missing	6	4.9	0	0	6	13.6	0	0	
Ethnicity parents^a^, (%)									
Both Caucasian	96	78.7	26	92.9	21	50.0	49	94.2	ODD/CD < ASD = TD***
Caucasian and other	11	9.1	2	7.1	6	14.2	3	5.8	
Both are unknown	4	3.3	0	0	4	9.5	0	0	
Highest level of education parents^b^, (%)									
Lower	7	5.7	0	0	4	9.5	3	5.8	
Middle	38	31.1	3	10.7	15	35.7	20	38.5	
Higher	63	51.6	25	89.3	10	23.8	28	53.8	ODD/CD < ASD < TD*

*FSIQ* full-scale IQ, *ICU* callous–unemotional traits based on the inventory of callousness–unemotional traits. *YPI* youth psychopathic trait inventory. *RPQ* reactive and proactive questionnaire. *SCQ* social communication questionnaire. *TD* typical developing individuals, *ASD* autism spectrum disorder, *ODD/CD* oppositional defiant disorder/conduct disorder, *na*: not assessed, *ns* not significant

*p* value: *p < 0.05; **p < 0.01, ***p < 0.001

^a^Ethnicity parents: data based on two parents

^b^Highest level of education parents: data based on family level

The difference between the number of participants initially approached and the final inclusion in this eye-tracking study is considerable large. In many cases, participants with ODD/CD were not interested in participating in an extensive clinical study. Many had behavioral problems and were often not in a position to participate. There were restrictions to leave closed institutions or their personal situation did not allow participation. Here, one can think of the occurrence of violent and/or oppositional incidents, escape attempts, and (temporary) dysfunctional relationships with their caregivers.

### Inclusion and exclusion criteria for participation

All participants who were recruited from clinical institutes obtained a clinical ASD or ODD/CD diagnosis prior to the study. Clinical diagnoses (ODD/CD and ASD) were established according to the DSM-IV-TR criteria [5] by a multidisciplinary team (experienced psychiatrist and psychologist). In a large proportion of our ASD participants, the clinical diagnoses were confirmed by clinical scores on the ‘golden standard’ of the ADOS and ADI, although this was not a fixed criterion for inclusion in this study.

They both gathered information and reviewed (prior) clinical records and information provided by schools and other agencies involved in the care of the adolescent. This workflow ensured that the proper clinical diagnosis was confirmed, before individuals were included in the current study. This is a robust and more reliable approach compared to only using structured interviews for the allocation of individuals to clinical groups [54].

For all the three groups, caretakers (i.e., biological parents or legal guardians) were asked to fill out a digital version of the National Institute of Mental Health Diagnostic Interview Schedule for Children (DISC-IV; [77]). Parents and/or caregivers had to complete the following sections of the DISC-IV: Attention-Deficit/Hyperactivity Disorder, ODD, CD, Tic Disorder, alcohol, marihuana, and other drug use. The social communication questionnaire (SCQ) was used as an instrument to assess ASD characteristics across the three groups (ASD, ODD/CD, and TD). For the typically developing group, the absence of a clinical psychiatric diagnosis was assessed based on the DISC-IV parent interview [77]. The outcomes of the DISC-IV and the SCQ were evaluated by an experienced child and adolescent psychiatrist (PH) and psychologist (MJB).

We excluded participants who fulfilled one or more of the exclusion criteria (a) a combined diagnosis of ASD and CD/ODD, (b) an estimated total IQ < 80); and/or (c) suffering from a condition which may affect neurological or cognitive functioning, such as schizophrenia, bipolar disorder, alcohol and/or drugs dependency, language disorder (e.g., dyslexia), epilepsy, and the presence of tics. The TD individuals were not allowed to have a clinically established psychiatric diagnosis to participate. The other in- and exclusion criteria were the same as for the clinical groups. Participants with a diagnosis of ODD or CD from the CU2 project were grouped together in this study, because both disorders are on a spectrum of behavior problems and aggressive tendencies. In addition, the ODD/CD group included only a few CD participants to be handled as a stand-alone group.

### Medication use

The use of non-psychotropic and anti-depressant medication was allowed for the inclusion in the study. If possible, psychotropic medication (i.e., antipsychotics, stimulants, and atomoxetine) was stopped prior to testing. Stimulants were discontinued for at least 24 h prior to participation and antipsychotics for at least 72 h. Only in cases, when a health care professional judged the discontinuation to have potential severe detrimental effects, the medication was not stopped. In total, 9 participants with ODD/CD and 8 participants with ASD were still on medication during the testing days.

### Cognitive assessments

Participants were required to have a minimum average estimated total full-scale intelligence quotient (FSIQ) IQ of ≥ 80. The FSIQ was estimated using four subtests of the Dutch version of the Wechsler Intelligence Scale for Children (WISC-III): Similarities, Block Design Picture Completion, and Vocabulary [85]. These WISC-III subtests are known to be highly correlated (*r* = 0.90–0.95) with full-scale IQ [40]. For the participants that were 16 years or older, the Wechsler Adult Intelligence Scale III (WAIS-III) was administered [86].

### Procedures

A short telephone screening and, subsequently, screening questionnaires were used to verify if families could participate. Those families were invited to visit one of the participating clinics. Testing of the participants took place in a quiet room at the test location. Experimenters used stimulus deprived rooms to limit the influence of distraction. Participants were given short breaks and received a financial compensation (vouchers of € 20.00) for this test administration.

### Ethical approval

This study was approved by the Dutch Central Committee on Research involving Human Subjects, protocol number NL26773.000.09 (Centrale Commissie Mensgebonden Onderzoek; CCMO). Both adolescents (if 12 years of age and older) and their legally appointed guardian provided written informed consent.

### Description of clinical measures

#### Social communication questionnaire (SCQ)

The social communication questionnaire is a 40-item parent-report questionnaire that investigates ASD characteristics on a binary scale (yes/no). The questionnaire contains 19 items on current behavior and 20 items on the period when the child was 4–5 years old [75]. A cut-off score of > 10 was used as a positive screening outcome on ASD characteristics. TD participants could only be included when they did not have a clinical score on the parent-rated SCQ (i.e., raw scores of < 10). In calculating the total score, the first item was excluded, because it only probed for sufficient language ability. The English version of the SCQ has a sensitivity ranging between 0.85 and 0.88 and a specificity between 0.72 and 0.78 [8, 19, 20]. The Cronbach’s alpha for the total SCQ score was 0.75 in the final sample.

#### Inventory of Callous–Unemotional traits (ICU)

The Inventory of Callous–Unemotional traits (ICU) assesses CU traits in adolescents, divided into three subscales: uncaring, callousness, and unemotional [37]. We used the official Dutch translated version of this questionnaire. Internal consistency of the Dutch ICU was shown to be good [34, 74]. The ICU exists of 12 positively framed items and 12 negatively framed items. Items are rated on a 4-point scale ranging from 0 (‘not at all true’) to 3 (‘definitely true‘). The uncaring scale consists of 8 items, the callousness subscale of 11 items, and the unemotional subscale of 5 items. An example of an item on the uncaring scale is ‘I am concerned about the feelings of others’. An example item for the callousness scale is: ‘I seem very cold and uncaring to others’. Finally, an example of the unemotional scale: ‘I do not show my emotions to others’. Subscale scores are calculated by summing the individual item scores. The reverse and ‘opposite’ framing of sentences is taken into account in the scoring. Subsequently, the total score is calculated by summing up the subscale scores. A higher total score reflects a higher levels of CU traits. We administrated both the parent version (legal guardian) and the self-rated version of the ICU. For the final sample, the Cronbach’s alpha for the self-report was 0.78 and the Cronbach’s alpha for the parent report was 0.90.

#### Youth psychopathic trait inventory (YPI)

The youth psychopathic traits inventory (YPI) is a 50-item self-report questionnaire [2]. It has been designed to assess core psychopathic personality traits for adolescents of 12 years of age and older. It reflects 3 dimensions of psychopathy: the grandiose manipulative, callous–unemotional, and impulsive–irresponsible [24]. Higher YPI total scores reflect the presence of high psychopathic traits. Internal consistency has been reported as 0.94 Cronbach’s alpha for the total score of the YPI, 0.82 for the grandiose–manipulative subscale, 0.64 for callous–unemotional subscale, and 0.76 for impulsive–irresponsible subscale.

#### Reactive and proactive aggression questionnaire (RPQ)

The Reactive and Proactive Aggression Questionnaire (RPQ) was developed by Raine et al. [71]. In the current study, the Dutch translation of the well-validated 23-item RPQ was used which is designed to probe for reactive and proactive aggression in children and adolescents from the age of 8 years of age and older [22]. The reactive subscale has 11 items. Example questions include: ‘He/she gets mad or hit others when they tease him/her’ and ‘He/she damages things when he/she is mad’. The proactive subscale has 12 items. Example questions for this subscale are: ‘He/she damages or breaks things for fun’ and ‘He/she threatens and bullies other kids’. The questions of the RPQ do not reference to a certain time period in the past or current behavior. Participants just have to report how often they have engaged in particular behaviors. The total score of the RPQ is calculated by summing all items together. The Cronbach’s alpha for the RPQ was 0.91 in our final sample.

### General study protocol

Participants and their legal guardians that gave informed consent were screened using the DISC-IV interview device [77]. The information of the DISC-IV was combined with the clinical diagnosis information to allocate participants into the different groups. The participants and their legal guardians were asked to fill out questionnaires (paper and pencil) separately from each other. This could either be at home or at the test location. For the test location, experimenters used a stimulus deprived quiet room. The influence of external noise and distraction was limited. For completing the questionnaires at home, the participants and their legal guardians were asked to sit in a quiet room with as few external distractions as possible.

### Task design

We used an emotional recognition task that consisted of 60 trials with static images of emotional and neutral faces. Each trial always had the same structure: ‘fixation cross (1 s)–facial stimulus (6 s)–question—gray screen (3 s)’. The rationale behind the presentation of the gray screen was twofold. First, we wanted to avoid the confound of pupil response to the differences in light intensity of the facial stimuli (presented on a black background) and the questions (presented on a white background). Second, the use of the gray screen countered potential ‘wash over effects’ of gazing at emotional faces and neutral faces and vice versa.

Trials with emotional faces and neutral faces were interleaved. The whole task consisted of two sessions of 30 trials that was interrupted by a short break. Both the sessions had a different order of the presentation of the emotional and neutral faces. All used faces were balanced on gender, ethnicity, and in the adult age range. A set of face stimuli were selected from the online NimStim of Facial Expressions set (available to the scientific community at http://www.macbrain.org/resources.htm) [81]. The faces differed on the intensity of portrayed emotion from high to low. Both the types of emotion and the portrayed intensity have been previously validated [42]. The Dutch question asked to the participants was presented on the screen and can be translated as ‘What kind of emotion did you see?’. The participants always had five answer options: neutral, happy, sad, angry, and fear. The order of the answers on the screen was balanced over the trials.

### Data pre-processing

We exported the fixation data from Tobii studio 2.2.08 and used Matlab 2016B [58] to pre-process the eye-tracking data. We used stringent data exclusion and inclusion criteria. Trials were excluded if there was no fixation data for 25% or more of the trial duration. At least 1.5 s of the 6 s trial duration had to contain valid eye-tracking data. To overcome and counter potential artifacts, we did not take the first 100 ms of the trail into account for the time to first fixation. In the first 100 ms, it is hard to disentangle ‘real fixations’ from potential measurement artifacts or limitations of the used apparatus with sampling rate of 50. Moreover, we excluded participants in which 50% or less of the trials were valid. Applying these criteria led to the exclusion of 4 participants (2 participants with ASD and 2 with ODD/CD).

### Normality of distributions

We investigated the distributions of all our eye-tracking output variables and checked for violations of normality. We used skewness and kurtosis to establish normality values (see supplementary Table 1 for more information). For all three groups and all the eye-tracking variables, normality could not be completely assumed. This led to the choice to use non-parametric statistics such as the Kruskal–Wallis tests [18], non-parametric Mann–Whitney post hoc tests, and Spearman correlations. The Spearman correlations are rank order free and resistant to violations of normality assumptions. In this way, we could ensure that eye-tracking variables in milliseconds would still have biological plausible meaning.

### Statistical analysis

**Fig. 1:**
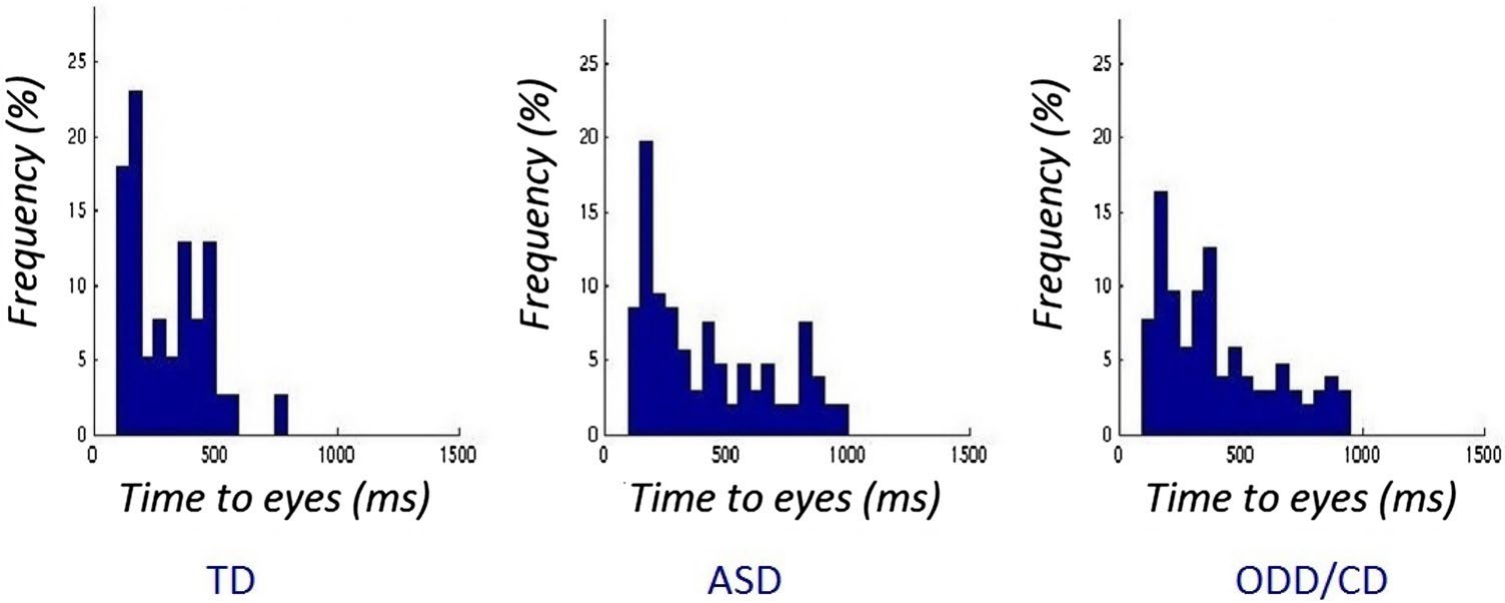
Distributions of the time to first fixation on the eyes of fearful faces for 100–1000 ms. Timebins are 50 ms each. *TD* typically developing individuals, *ASD* autism spectrum disorder, *ODD* oppositional defiant disorder, *CD* conduct disorder

We applied a non-parametric trial-based approach to investigate gazing behavior on the AIO (eyes, mouth, and rest of the image) of emotional and neutral faces [18]. For all our three main eye-tracking variables (total fixation duration, time to first fixation, and percentage total fixation), we used Kruskal–Wallis one-way ANOVA tests (two-tailed, significance level *α* = 0.05) to test for group differences. For the variable ‘time to first fixation’ on the eye AIO, we investigated the relative distribution (percentagewise) for all three groups (ASD, ODD/CD, and TD) for values in time bins of 50 ms. To overcome and counter potential artifacts, we did not take the first 100 ms of the trial into account for the time to first fixation. In the first 100 ms, it is hard to disentangle ‘real fixations’ from potential measurement limitations of the used apparatus. We ran five tests separately for all the different emotions (anger, sad, fear, and happiness) and neutral faces (see Fig. 1). We applied Bonferroni corrections for multiple testing (two-tailed, significance level, *α* = 0.01). We performed Mann–Whitney post hoc tests to examine the specific directionality of effects between the groups. The same rationale was followed for all of our eye-tracking variables. For the investigation of behavioral results of the emotion recognition task, we looked at the percentages of correct answers per group and tested for group differences via *t* tests after *z* score transformations.

Furthermore, we investigated the correlation (Spearman test, two-tailed, significance level *α* = 0.05) between eye-gazing pattern for emotional faces with CU traits (total score of ICU, and YPI CU subscale scores), psychopathic traits (YPI total score), and severity of aggression (RPQ scores for proactive and reactive aggression).

## Results

### Descriptive results

See Table 1 for sample characteristics. The three groups did not differ in age, but significantly differ in estimated full-scale IQ (FSQ). A post hoc test revealed that the FSQ was lowest for the ODD/CD group, and highest in the TD group, while the ASD group scored in between of the two. There was no significant difference between the ASD group and the TD group. The three groups significantly differed on SCQ, post hoc test revealed that the ASD group scored higher than the ODD/CD group. Regarding *self*-rated CU traits, the ODD/CD group showed significantly higher CU scores than both the TD and ASD groups, whereas the TD and ASD groups did not differ from each other. Regarding the parent-rated CU traits, the ODD/CD group scored significantly higher than the ASD group, and the ASD group scored significantly higher than the TD group. The three groups differed significantly from each other on aggressive behavior (RPQ total score). A post hoc test showed that the ODD/CD group had significantly higher scores on the RPQ total score then both the ASD and the TD groups. The ASD group did not differ from the TD group. Regarding reactive aggression, the ODD/CD group had significantly higher scores than both the ASD and TD groups. The ASD group and the TD group did not differ from each other. Regarding proactive aggression, the ODD/CD group had significantly higher scores than both the ASD and TD groups. The ASD group and the TD group did not differ from each other (Table 2).

**Table 2:** Eye-tracking results for gazing at the eyes

Total fixation duration	*N*	Degrees of freedom	Chi square	Significance	Contrasts post hoc test
Anger	774	2	511.5	*p *= 0.003	TD > ODD/CD**
Fear	835	2	15.1	*p *< 0.01	TD > ODD/CD**
Sad	816	2	0.6	*p *= 0.74	ns
Happy	835	2	15.2	*p *= 0.001	
Neutral	2866	2	31.2	*p *< 0.001	TD > ASD*** TD > ODD/CD***
Time to first fixation					
Fear	248	^2^	6.11	*p *= 0.047	TD > ASD = ODD/CD*
Anger	222	2	4.04	*p *= 0.1	ns
Sad	234	2	2.47	*p *= 0.5	ns
Happy	264	2	5.79	*p *= 0.055	ns
Neutral	116	2	3.25	*p *= 0.19	ns

*TD* typically developing individuals, *ASD* autism spectrum disorder, *ODD* oppositional defiant disorder, *CD* conduct disorder

*ns* not significant, *p < 0.05; **p < 0.01, ***p < 0.001

### Eye-tracking results and behavioral results

**Fig. 2:**
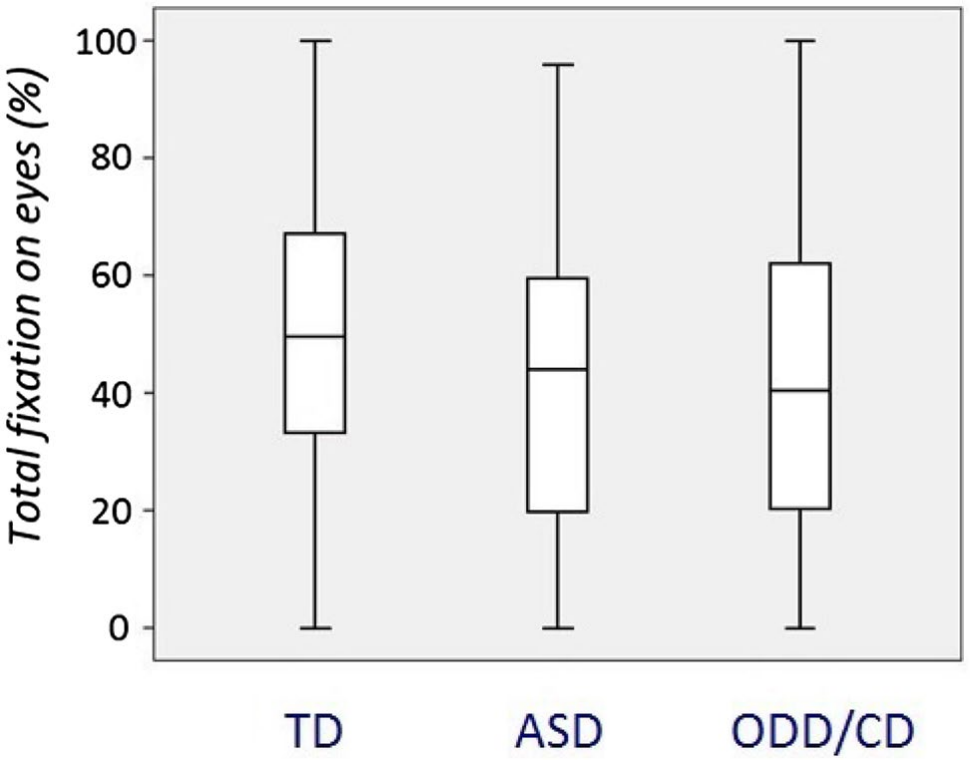
Percentage of total fixation duration on the eyes of fearful faces. *TD* typically developing individuals, *ASD* autism spectrum disorder, *ODD* oppositional defiant disorder, *CD* conduct disorder

We found a main group effect for relative total fixation time to the eye region for fearful (Kruskal–Wallis one-way ANOVA, [*χ*^2^ (*df* = 2, *N* = 835) = 15.1, *p *< 0.01] (presented in Fig. 2), angry [*χ*^2^ (*df* = 2, *N* = 774) = 511.5, *p* < 0.01], happy [*χ*^2^ (*df* = 2, *N* = 835) = 15.2, *p *= 0.001], and neutral faces [*χ*^2^ (*df* = 2, *N* = 2866) = 31.2, *p *< 0.001]. The *N* number is representing the number of trials per emotion per experimental group. When correcting for multiple comparisons via Bonferroni correction (*p *= 0.05/5 = 0.01), these main effects remained significant. Mann–Whitney post hoc tests revealed that the TD group had significantly more fixations to the eye region than the participants with ASD or ODD/CD for fearful, angry, happy, and neutral faces, whereas the ASD and ODD/CD groups did not differ from each other. There was no main group effect for sad faces [*χ*^2^ (df = 2, *N* = 819) = 0.6, *p *= 0.7].

**Fig. 3:**
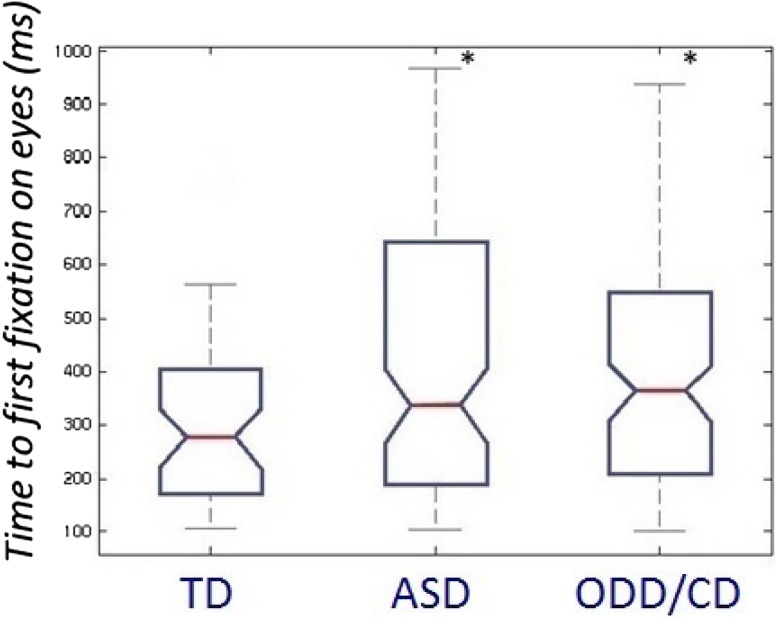
Time to first fixation in milliseconds on the eyes of fearful faces. *TD* typically developing individuals, *ASD* autism spectrum disorder, *ODD* oppositional defiant disorder, *CD* conduct disorder

We found a main group effect for the time to first fixation towards the eye region for fearful faces [Kruskal–Wallis one-way ANOVA, *χ*^2^ (*df* = 2, *N* = 248) = 6.11, *p* = 0.046] (Fig. 3). When correcting for multiple comparisons via Bonferroni (0.05/5 = 0.01), this main group effect did not survive.

We performed Mann–Whitney post hoc tests to investigate the directionality of this nominal significant main effect. That revealed that both groups with ASD or ODD/CD took significantly longer time to first fixate on the eyes of a fearful face, compared to TD participants. We did not find any main group effects on time to first fixation to the eye AIO for sad, angry, happy, and neutral faces.

The behavioral results for the emotion recognition task are presented in Table 3. We looked at the percentages of correct answers and the group differences via *t* tests via normalized *z* scores. We found significant differences between the ODD/CD group and the TD group for the happy faces (*p *< 0.005) and sad faces (*p *= 0.01). We also found differences between the ODD/CD group and the ASD group for neutral faces (*p *< 0.05), sad faces (*p *= 0.03), and fearful faces (*p *= 0.02). These are all nominal significants, since only the result for happy faces survives Bonferroni correction (0.05/5 = 0.01).

**Table 3:** Behavioral results of emotion recognition task

Emotions	TD	ODD/CD	ASD	Contrast	Significance
Neutral	69.3	66.1	63.2	ODD/CD—TD*	*p *= 0.046
Angry	46.6	45.5	41.0		
Happy	96.1	91.7	90.3	ODD/CD—TD***	*p *= 0.005
Sad	59.9	52.1	46.1	ODD/CD—TD* ODD/CD—ASD*	*p *= 0.01 *p *= 0.03
Fearful	84.0	80.6	75.2	ODD/CD—ASD*	*p *= 0.02

Depicted are the percentages correctly recognized emotional faces The effects are bases on *t* tests, normalized with z-transformation

*TD* typically developing individuals, *ASD* autism spectrum disorder, *ODD* oppositional defiant disorder, *CD* conduct disorder

*ns* not significant, *p < 0.05; **p < 0.01, ***p < 0.001

### Correlations of eye-tracking variables and behavioral traits

**Fig. 4:**
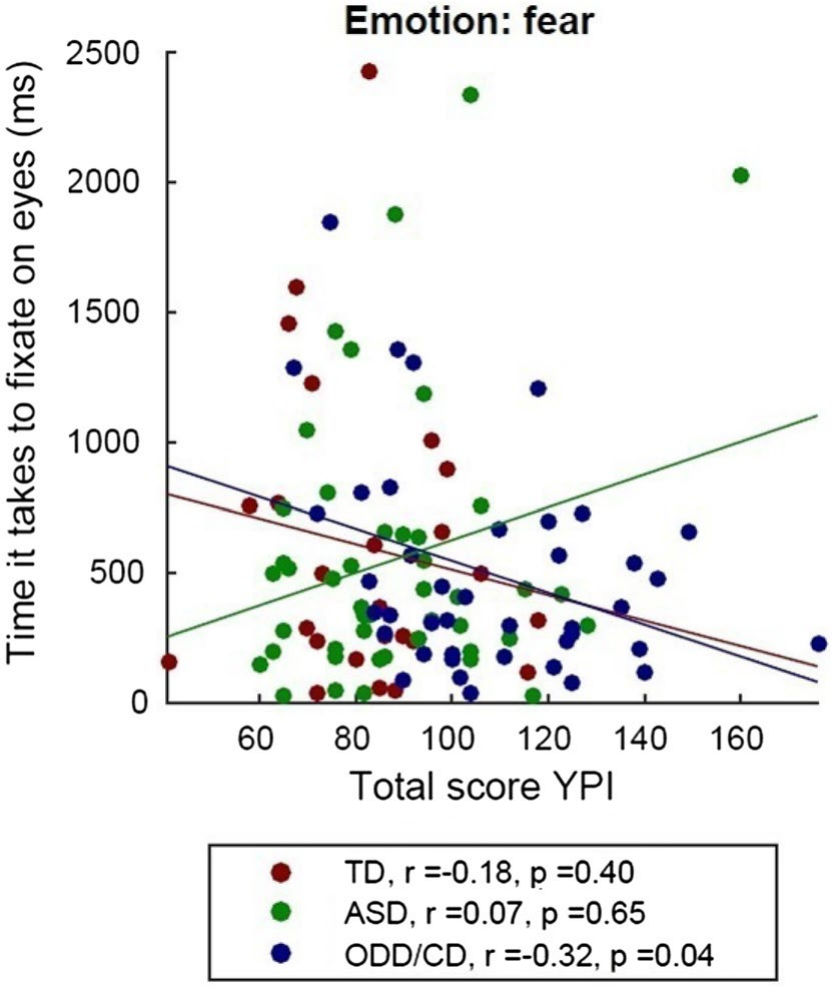
Spearman correlations between the time to first fixation on the eyes of fearful faces and total score of the YPI. *TD* typically developing individuals, *ASD* autism spectrum disorder, *ODD* oppositional defiant disorder, *CD* conduct disorder

**Fig. 5:**
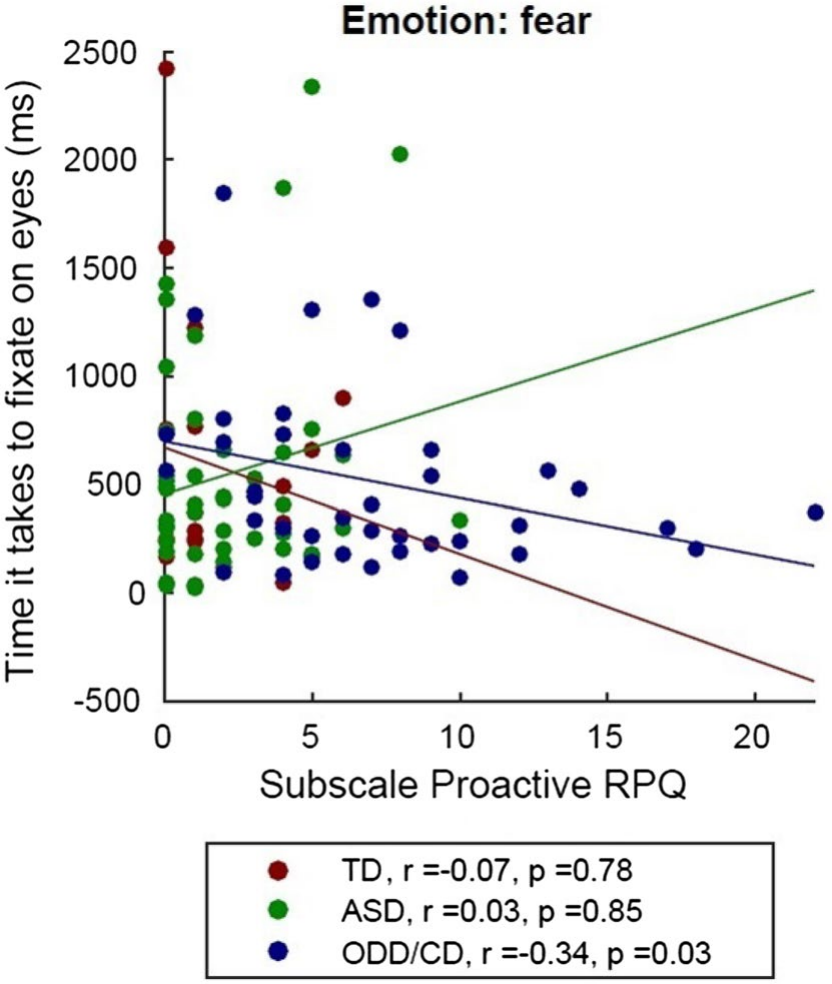
Spearman correlations between the time to first fixation on the eyes of fearful faces and proactive aggression (RPQ- proactive subscale). *TD* typically developing individuals, *ASD* autism spectrum disorder, *ODD* oppositional defiant disorder, *CD* conduct disorder

Only in the ODD/CD group, we found a nominal significantly negative Spearman correlation between the time to first fixation at the eyes of fearful faces and psychopathic traits (*r* = 0.35, *p* = 0.02) (Fig. 4). When correcting for multiple comparison via Bonferroni correction (*p *= 0.05/5 = 0.01), the Spearman correlation did not survive this correction. In addition, proactive aggression was also negatively correlated (*r* = − 0.33, *p* = 0.04) with time to first fixation to the eyes of fearful faces in the ODD/CD group. When correcting for multiple comparison via Bonferroni correction (*p* = (0.05/5) = 0.01), this correlation also did not survive (Fig. 5).

For the other three emotions; happiness, sadness, anger, and neutral faces, the psychopathic traits, CU traits, and aggressive tendencies did not correlate with any of the eye-tracking variables on any of the AOIs.

### Control analyses

To check if our effects were not driven by the known significant differences in intelligence, ADHD comorbidity and medication use between groups, we undertook additional analyses. We regressed out full-scale intelligence (FSQ) from the model. The Spearman correlation between the total YPI score and the time to first fixation on the eyes was not significant anymore (*r *= 0.26, *p *= 0.14). For the ASD group, the correlation was significant (*r *= 0.31, *p *= 0.04), but did not survive Bonferroni correction (*p *= 0.05/5 = 0.01). The Spearman correlation between proactive aggression and time to first fixation on the eyes of fearful faces was not significant anymore (*r *= 0.18, *p *= 0.3). Concluding, the regression of full-scale intelligence scores from the model did not change the directions of the effect and all Spearman correlation still did not survive correction for multiple comparison. Furthermore, we ran analyses to control for the effects of ADHD comorbidity and medication use (for details, see supplementary Tables 2, 3, 4, and 5). In the sample, 50 (28 subjects with ODD/CD and 22 subjects with ASD) of the 122 subjects had a comorbid ADHD diagnosis, and in the case of 9 participants, information was missing. For both control analyses, we excluded those participants with either ADHD (or missing information) or on medication (or missing information).

The directions of the effects in both control analyses did not differ from the effects in the main analysis for total fixation duration, relative total fixation duration, and time to first fixation. Moreover, we ran control analysis for the Spearman correlation with behavioral traits (psychopathic traits and proactive aggression). In this case, we only selected participants without ADHD or that were not using medication (or missing information). The direction of the effects between the time to first fixation on the eyes of fearful faces in the ODD/CD group and psychopathic traits (YPI total score) and proactive aggression (RPQ proactive aggression subscale) did not change (see supplementary Table 5 for these results).

## Discussion

This study aimed to investigate common cross disorder and unique disorder-specific patterns of eye gaze during emotional face processing by a head-to-head comparison of male adolescents with either ASD, or ODD/CD, compared to TD for eye-tracking measures (1) time to first fixation on an AIO, (2) total fixation duration to an AOI, and (3) percentage of total fixation duration on an AOI relative to the rest. We also examined the modulating role of CU and psychopathic traits, and aggression subtypes. We chose not to include subjects with a combined diagnosis of both ASD and ODD/CD to facilitate a clear cross-disorder comparison. In this way, we are not looking at the combined comorbid group (with a diagnosis of both ASD and ODD/CD) and cannot compare synergistic effects arising from the comorbidity of these disorders. Our results showed that (1) participants with either ASD or ODD/CD both did fixate proportionally and significantly less on the eye region of emotional faces (with sadness excepted) and neutral faces, compared to TD. (2) participants with either ASD or ODD/CD both took longer time to first fixation on the eye region in fearful faces, but not in faces with the other emotions/emotional expressions (i.e., neutral, anger, sadness, and happiness). However, this effect did not survive multiple comparison correction. (3) When looking at the relationship between eye gazing and CU traits, psychopathic traits, and aggression, we found a seemingly opposing effect. Higher scores for psychopathic traits and of proactive aggression within the ODD/CD group were nominally significant associated with shorter time to first fixation at the eye region for fearful faces compared to the TD group. All three groups were paying more attention to the eye region compared to the mouth region and other parts of the face. Since these effects did not survive multiple comparison and regressing out full-scale intelligence scores did not change this, we did not find solid evidence for the hypothesized relationships.

Some studies have indicated that excessive attention to the mouth region may be adaptive for ASD children with well-developed language skills [72]. More recent work falsifies the gaze aversion to the eyes in infants [59]. These findings are not confirmed in our high-functioning adolescent male population with ASD. The differential results can be explained by differences in methodology across laboratories and also the high heterogeneity in gazing behavior for individuals with ASD. The different age ranges of samples and their intelligence profiles may also partly explain differences in findings [41].

Earlier studies reported poorer recognition of emotional facial expressions in individuals with CD [36, 87] and also abnormally low amygdala activations to fearful or angry emotional faces in individuals with CD, particularly those with high CU traits [50, 67]. We observed both in the ODD/CD and ASD groups proportionally less gazing at the eye region of emotional and neutral faces. This suggests that less gazing at the eye region of emotional faces might still be a cross-disorder trait that is not unique to ASD, but shared with other disorders like ODD/CD, which is in line with findings that emotion recognition problems characterize a wide range of child psychiatric disorders, varying from ASD, ADHD, and CD to mood and anxiety disorders and eating disorders and schizophrenia [23].

The novelty of our study is that we are providing insight into differences of eye-gazing behavior on fearful faces between clinical groups that are well-phenotyped and look at the links with psychopathic traits, CU traits, and aggression. We found that the time to first fixation is delayed for the ODD/CD group for the time to first fixating on the eyes of fearful faces. A delayed first fixation to fearful eyes might lead to slower processing and delayed evaluating of the fearful state of the other person. Small distortions in synchrony of emotional communication between individuals due to delayed processing of emotional information may already disrupt social interactions and predispose to inadequate and even harmful behavior [14, 53].

There was a nominally significant negative correlation in our ODD/CD group between the time to first fixation to the eyes of fearful faces and psychopathic personality traits (YPI). This effect did not survive multiple comparison correction. This dimensional effect concerning higher psychopathic traits is opposite the group effect of eye gazing in our ODD/CD participants that gaze later to the eyes of fearful faces. The absence of a relationship between psychopathic/CU traits and gaze fixation in both the ASD group and the TD group might be due to the smaller variance in psychopathy and CU scores (for details, see Table 1) in these groups. It might be that a selection bias led to the oversampling of participants lower than average on psychopathic traits, CU traits, aggression for those that score average or high might be less willing to be subjected to testing in a clinical research setting.

In general, a modulating role of psychopathic traits is consistent with findings in functional MRI studies, where amygdala activation to fearful or angry faces is low in the presence of high psychopathic and high in their absence [50, 67]. Klapwijk et al. [51] also found decreased amygdala responses in both adolescents with ASD and individuals with CD and high CU traits. We also found a possible association with the severity of in particular proactive aggression and time to first fixation on the eyes of fearful faces. Children as well as adolescents and adults with ODD/CD and high levels of psychopathy/CU traits are more likely to have high levels of proactive aggression [26, 39]. Our data does seem to suggest a potential link between ODD/CD, high psychopathic traits, proactive aggression, and impaired fear processing.

Although we document similar patterns of abnormal gaze behavior to emotional faces in ASD and ODD/CD, the underlying mechanism might be disorder specific. There are three theories trying to explain abnormal emotional face processing in ASD. First, gazing at faces and eyes in particular may lead to increased (negatively valence) emotional responses in individuals with ASD and even found to be aversive [31]. Looking at the mouth is then just a byproduct of avoiding gazing at the eyes. Second, another theory poses that individuals with ASD cannot “read the language of the eyes”, i.e., they do not understand visual information from the eyes which may be linked to problems in using a Theory of Mind [52]. The failure to use information from the eye region in combination with an ability to use visual information from the mouth for speech related processing is driving the deficit of excess fixation on the mouth and diminished fixation on the eyes. Third, another explanation is that individuals with ASD are suffering from impaired social orientation and that the “most social” part of the face, the eye region lacks saliency and does not arouse sufficient intrinsic interest to be looked at [41]. Unfortunately, our paradigm and our results not allow us to differentiate between these potential explanations.

Impaired affective responses and emotional processing in CD has been addressed by three main theories [32]. The attention to the eyes hypothesis proposes that emotion processing deficits in CD/psychopathy arise from a lack of spontaneous attention to the eye region [30, 28] which negatively affect the processing of all emotional expressions. The distress-specific hypothesis states that individuals with CD/psychopathy fail to effectively process in particular others’ expressions of distress (fear and sadness). As a result, their antisocial actions are not inhibited by aversive feelings of remorse and guilt, resulting in callous behavior and shallow affect [15, 11]. Finally, the enhanced selective attention hypothesis [63, 61, 62] states that the enhanced ability to focus on a task and to ignore goal irrelevant stimuli underlies affective deficits. This superior selective attention can enhance the top–down ability to suppress emotional information that is irrelevant to one’s goals, for example, another person’s distress if the psychopath wants to steal their money. Since the gaze pattern with proportionally less attention to the eye region was observed for all emotions except for sadness, our results are mostly in line with the attention to the eye hypothesis or the enhanced ability to focus hypothesis.

Our groups did not differ with respect to the total fixation duration on the eyes while processing sad faces. Other studies have shown that emotional recognition deficits for sadness are present in people with ODD/CD [79, 87]. These discrepant findings may be due to differences between studies in sample selection and characteristics. The Woodworth and Waschbusch [87] sample consisted of both male and female children with high levels of CU traits, which is quite different from our male adolescent sample. The Stevens et al.’s [79] sample did not use a formal clinical diagnosis of ODD or CD and selected participants on the basis of a score of 25 or higher on the psychopathy screening device [38].

Despite its strengths, such as the direct comparison of a well-powered ASD and ODD/CD group and its focus of gaze behavior by means of eye-tracking measures which ruled out the influence of social desired expected answers of questionnaires, our study also showed limitations. We were not able to control the gaze duration to the fixation cross prior to the faces that were portrayed on the screen. As half of our trials contained neutral faces, we did not have enough trials per emotion to look into the effects of gender, ethnicity, and the intensity effects of the emotions portrayed on the faces. The stimuli used in this study were selected from a validated database of emotional expressions: including stimuli with facial characteristics such as wrinkles and facial hair. Facial characteristics can be seen as a factor that may influence the study outcomes. In contrast to studies that use morphed faces, our facial stimuli are closer to emotional faces in the real world. On the other hand, this might potentially revert the attention of the participants and confound the outcome. Both diagnostic groups also contained a substantial amount of participants with comorbid ADHD and/or using medication. Although antipsychotics (where possible) were stopped 2 days before, and stimulants on the test day, we cannot rule out possible medication effects. However, sensitivity analyses revealed that results were not influenced by the presence of a comorbid diagnosis of ADHD or by medication use.

### Implications

Considering the consequences of aggression, there is a need for a better understanding of underlying causes and maintaining factors. The current study contributes to the enhancement of this understanding by revealing (1) two cross-disorder traits for ASD and ODD/CD; (2) disorder-specific traits for ODD/CD with proactive aggression as a potential factor. Future research is warranted to examine possible other cross-disorder traits (e.g., biological and genetic) and/or disorder-specific traits; and (3) adding to knowledge and understanding in fractioning empathy to emotional stimuli by means of eye-gazing processing as a part of the MATRICS project (http://matrics-project.eu/). MATRICS examines the neural, genetic, and molecular factors involved in the pathogenesis of aggression/antisocial behavior and that in relation with callous–unemotional traits.

Moreover, as the current treatments, which mainly involve skill training, are not suitable or developed to alter implicit characteristics, other methods are needed to improve the efficacy of aggression treatment, and techniques like virtual reality seem to be promising [66, 69]. Clinical implications are mainly optimization of psychological interventions by therapists requiring eye-gazing information. A future study would definitely also benefit from the presentation of both static and dynamic faces as stimuli and comparing outcomes.

## Conclusions

To conclude, we reported that male adolescents with ASD or ODD/CD looked less at the eyes in fearful, angry, happy, or neutral emotional expressions. They also took nominal significantly more time to first fixate on the eyes of fearful faces compared to TD. Those male adolescents with ODD/CD that exhibit faster first fixations on the eyes of fearful faces had nominal significant higher scores on psychopathic traits. Nevertheless, we did not find strong evidence that survived multiple comparisons to support that in ASD and ODD/CD higher scores on CU traits, psychopathy, and aggression were related to eye gazing on the eyes of fearful faces. Our data do provide valuable and new insight into the gaze behavior distributions of ODD/CD and ASD groups when looking at the eyes of emotional faces.

## Electronic supplementary material

Below is the link to the electronic supplementary material.

Supplementary material 1 (DOCX 23 kb)
