# Supplementary material for: Emotional face recognition in male adolescents with autism spectrum disorder or disruptive behavior disorder: an eye-tracking study
Source: Eur Child Adolesc Psychiatry. 2018 Jun 19;27(9):1143–57. doi: 10.1007/s00787-018-1174-4 (PMC6133091; doi:10.1007/s00787-018-1174-4)
Supplement: Supplementary file 1 — Supplementary material 1 (DOCX 23 kb) [file 787_2018_1174_MOESM1_ESM.docx]

**Supplementary table 1. Normality indices of eye tracking variables**

|  | **All subjects - skewness** | **All subjects - kurtosis** | **ASD group - skewness** | | **ASD group - kurtosis** | **ODD/CD group - skewness** | **ODD/CD**  **Group - kurtosis** | **TD**  **group**  **skewness** | **TD group -**  **Kurtosis** |
| --- | --- | --- | --- | --- | --- | --- | --- | --- | --- |
| **Fixation duration** | | | | | | | | | |
|  | -1.34 | 5.79 | -2.40 | 10.56 | | -0.52 | 2.74 | -0.63 | 2.37 |
| Fear | -0.95 | 4.42 | -0.69 | 3.58 | | -1.10 | 4.83 | -0.72 | 2.34 |
| Sad | -1.20 | 5.23 | -1.50 | 6.21 | | -0.91 | 3.78 | -0.55 | 2.46 |
| Happy | -1.30 | 5.66 | -1.99 | 9.24 | | -0.82 | 3.15 | -0.73 | 2.90 |
| Neutral | -0.05 | 2.57 | -0.07 | 3.07 | | 0.10 | 2.51 | -0.22 | 2.00 |
| **Time to first fixation** | | | | | | | | | |
| Fear | 2.96 | 10.65 | 3.41 | 14.95 | | 2.86 | 9.85 | 2.26 | 4.83 |
| Anger | 3.35 | 12.75 | 3.85 | 19.51 | | 3.12 | 10.20 | 3.09 | 10.47 |
| Sad | 3.29 | 12.7 | 2.88 | 9.99 | | 3.46 | 13.64 | 3.29 | 11.81 |
| Happy | 2.80 | 8.15 | 3.02 | 10.38 | | 2.48 | 5.70 | 2.94 | 8.67 |
| Neutral | 3.50 | 14.35 | 3.34 | 12.58 | | 3.46 | 13.64 | 3.53 | 15.25 |

TD: typically developing individuals; ASD: autism spectrum disorder; ODD: oppositional defiant disorder; CD: conduct disorder

**Supplementary table 2. Control analysis ADHD comorbidity**

| **Total fixation duration** | **N** | **Degrees of freedom** | **Chi-Square** | **Significance** | **Contrasts Post hoc tests** |
| --- | --- | --- | --- | --- | --- |
| Anger | 458 | 2 | 14.83 | *p* = .001 | TD > ODD/CD** |
| Fear | 461 | 2 | 21.01 | *p* < .001 | TD > ODD/CD* |
| Happy | 467 | 2 | 13.48 | *p* = .01 | TD > ODD/CD** |
| Neutral | 1575 | 2 | 51.50 | *p* < .001 | TD > ODD/CD* |
| **Time to first fixation** |  |  |  |  |  |
| Fear | 120 | 2 | 6.07 | *p* = .04 | TD = ODD/CD (n.s) |

n.s.: Not significant, * = p<.05; ** = p<.01, ***= p<.001

TD: typically developing individuals; ASD: autism spectrum disorder; ODD: oppositional defiant disorder; CD: conduct disorder

**Supplementary table 3. ADHD-comorbidity control analysis for the correlation with YPI and RPQ**

|  | **Group** | **Spearman (R)** | **Significance** |
| --- | --- | --- | --- |
| **Correlation time to first fixation - YPI** | | |  |
| Fear | TD | -.17 | *p* = .42 |
| Fear | ASD | .04 | *p* = .84 |
| Fear | ODD/CD | -.27 | *p* = .45 |
| **Correlation time to first fixation - RPQ proactive subscale** | | |  |
| Fear | TD | -.08 | *p* = .76 |
| Fear | ASD | -.10 | *p* = .63 |
| Fear | ODD/CD | -.63 | *p* = .08 |

* = *p* < .05, ** = *p* < .01, *** = *p* < .001

TD: typically developing individuals; ASD: autism spectrum disorder; ODD: oppositional defiant disorder; CD: conduct disorder

**Supplementary table 4. Control analysis for medication use during testing days**

| **Total fixation duration** | **N** | **Degrees freedom** | **Chi-Square** | **Significance** | **Contrasts post hoc tests** |
| --- | --- | --- | --- | --- | --- |
| Anger | 476 | 2 | 5.50 | *p* = .06 | TD = ASD (n.s.) TD > ODD/CD* |
| Fear | 487 | 2 | 6.76 | *p* = .03 | TD = ASD (n.s.) TD > ODD/CD* |
| Happy | 484 | 2 | 6.45 | *p* = .04 | TD = ASD (n.s.) TD > ODD/CD* |
| Neutral | 1653 | 2 | 8.27 | *p* = .02 | TD > ASD* TD > ODD/CD** |
| **Time to first fixation** | | |  |  |  |
| Fear | 120 | 2 | 6.07 | *p* = .04 | TD < ASD* TD = ODD/CD (n.s.) |

n.s.: Not significant, * = *p* < .05, ** = *p* < .01, *** = *p* < .001

TD: typically developing individuals; ASD: autism spectrum disorder; ODD: oppositional defiant disorder; CD: conduct disorder

**Supplementary table 5. Control analysis medication use during testing days**

|  | **Group** | **Correlation (Rs)** | **significance** |
| --- | --- | --- | --- |
| **Time to first fixation – YPI - total** | | |  |
| Fear | TD | -.18 | *p* = .4 |
| Fear | ASD | -.02 | *p* = .9 |
| Fear | ODD/CD | -.22 | *p* = .3 |
| **Correlation**  **Time to first fixation RPQ - proactive** | | |  |
| Fear | TD | -.07 | *p* = .78 |
| Fear | ASD | -.15 | *p* = .54 |
| Fear | ODD/CD | -.41 | *p* = .07 |

TD: typically developing individuals; ASD: autism spectrum disorder; ODD: oppositional defiant disorder; CD: conduct disorder

Description of Eye tracking metrics and methods

Apparatus

Stimuli and tasks were presented on a Tobii 1750 eye-tracking monitor (1280 x 1024 pixels), with built-in infrared eye-tracking sensors (Tobii Technology, 2006a). Eye position was measured as coordinates from both eyes continuously with a frequency of 50 Hz. We selected the raw data (x and y-coordinates, timestamps) as input for Tobii studio statistics (Tobii studio version 2.2.8, Tobii Technology, 2006b). The internal classification of fixations by Tobii studio was done based on a velocity-based approach. Internally, I-VT filters were used with a 1.75 velocity threshold in the case of our 50 Hz sampling (35/20=1.75). Eye-tracker calibration was performed before the task with a default 9-point calibration (Tobii Technology, 2006b). Participants were instructed to maintain a distance of approximately 60 centimeters from the eye-tracking monitor.

Areas of interest

The pictures that showed the faces had a pixel size of 768-1024 (20.32 - 27.1 cm). The eyes, mouth and rest of the images were predefined Areas Of Interest (AOI). The eye AOI was drawn as a rectangle that fitted the upper sides of the eyebrows, the extreme corners of the eye or brows and some space underneath the eyes. The mouth AOI was a fitted rectangle to the upper side of the mouth, the extreme corner of the mouth and some space underneath the mouth. Since we used real faces from a validated databank the AOI’s did not have exactly the same dimensions.

Eye tracking variables

One most cited eye-tracking variables is gaze fixation, defined as a set of consecutive gaze coordinates, confined within a diameter of 1◦ of visual angle for duration of 100 milliseconds or longer (Norton & Stark, 1971). We used three variables in our analysis that were all taken gaze fixations into account. 1) the total amount of fixations on an AIO (eyes, mouth and rest of the image). 2) The percentage total fixations on an AOI relative to the total amount of fixations. 3) The time to first fixation to an AOI. The first fixation specifies the time point in milliseconds from the start of the presentation of the face to the first fixation on the specific AOI of a face.
